# Application of informatics in cancer research and clinical practice: Opportunities and challenges

**DOI:** 10.1002/cai2.9

**Published:** 2022-06-15

**Authors:** Na Hong, Gang Sun, Xiuran Zuo, Meng Chen, Li Liu, Jiani Wang, Xiaobin Feng, Wenzhao Shi, Mengchun Gong, Pengcheng Ma

**Affiliations:** ^1^ Department of Medical Sciences Digital Health China Technologies Co., Ltd. Beijing China; ^2^ Xinjiang Cancer Center, Key Laboratory of Oncology of Xinjiang Uyghur Autonomous Region The Affiliated Cancer Hospital of Xinjiang Medical University Ürümqi China; ^3^ Department of Information, Central Hospital of Wuhan Tongji Medical College, Huazhong University of Science and Technology Wuhan China; ^4^ National Cancer Center, National Clinical Research Center for Cancer, Cancer Hospital Chinese Academy of Medical Sciences and Peking Union Medical College Beijing China; ^5^ Big Data Center, Nanfang Hospital Southern Medical University Guangzhou China; ^6^ Hepato‐Pancreato‐Biliary Center, Beijing Tsinghua Changgung Hospital School of Clinical Medicine, Tsinghua University Beijing China; ^7^ Institute of Health Management Southern Medical University Guangzhou China

**Keywords:** artificial intelligence application, cancer informatics, machine learning

## Abstract

Cancer informatics has significantly progressed in the big data era. We summarize the application of informatics approaches to the cancer domain from both the informatics perspective (e.g., data management and data science) and the clinical perspective (e.g., cancer screening, risk assessment, diagnosis, treatment, and prognosis). We discuss various informatics methods and tools that are widely applied in cancer research and practices, such as cancer databases, data standards, terminologies, high‐throughput omics data mining, machine‐learning algorithms, artificial intelligence imaging, and intelligent radiation. We also address the informatics challenges within the cancer field that pursue better treatment decisions and patient outcomes, and focus on how informatics can provide opportunities for cancer research and practices. Finally, we conclude that the interdisciplinary nature of cancer informatics and collaborations are major drivers for future research and applications in clinical practices. It is hoped that this review is instrumental for cancer researchers and clinicians with its informatics‐specific insights.

## Graphical abstract

This review summarized the progress of cancer informatics in the big data era. Various informatics methods and tools that are widely applied in cancer research and practices were discussed. This review further addressed the informatics challenges and opportunities for the comprehensive cancer field. It is expected that this review is instrumental for cancer researchers and clinicians with an informatics‐specific insight.

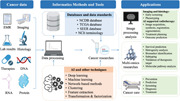

**Abbreviations**
AE: Adverse event
AI: artificial intelligence
CBCT: cone-beam computed tomography
CCTOO: The Cancer Care Treatment Outcome Ontology
CDC: Centers for Disease Control and Prevention
cGAN: conditional Generative Adversarial Network
CT: Computed Tomography
CTCAE: Common Terminology Criteria for Adverse Events
DCDB: National Cancer Database
DIR: deformable image registration
EMR: Electronic medical records
EPID: electronic portal imaging devices
ESR1: estrogen receptor 1
ICD-O: International Classification of Diseases for Oncology
ICGC: The International Cancer Genome Consortium
LYNA: LYmph Node Assistant
MedDRA: Medical Dictionary for Regulatory Activities
ML: machine learning
MRI: magnetic resonance imaging
MS: Mass Spectrometry
NCCR: National Central Cancer Registry
NCDB: The National Cancer Database
NCI: National Cancer Institute
NCIT: National Cancer Institute Thesaurus
NGS: next-generation sequencing
NPCR: National Program of Cancer Registries
PCO: Prostate Cancer Ontology
PET: positron-emission tomography
ROO: The radiation oncology ontology
ROS: The Radiation Oncology Structures
sCT: synthetic computed tomography
SEER: Surveillance, Epidemiology, and End Results
SNOMED CT: Systematized Nomenclature of Medicine Clinical Terms
TCGA: The Cancer Genome Atlas
TNM-O: TNM-Ontology
UMLS: Unified Medical Language System
WHO: World Health Organization

## 1 INTRODUCTION

Advances in information science and technology have brought significant benefits to cancer research and care, including larger study cohorts, more complete follow‐up, more effective clinician teams, lower costs, increased patient life expectancy, and improved quality of life. Despite all cancer‐related aspects, such as diagnosis, prognosis, and treatment being significantly improved, this disease area remains one of the most significant challenges in medical science due to disease heterogeneity and the need to identify underlying biomarkers that are potentially linked to specific cancer types.

Cancer informatics is a branch of medical informatics that applies information science, computer science, data science, and information technologies to the field of oncology. This is an area that deals with the resources, devices, and methods required to optimize the acquisition, storage, retrieval, and use of information in cancer. Applied cancer informatics transforms clinical data into meaningful and useful information to improve processes and outcomes in patient‐focused and evidence‐based cancer care [1]. The fundamental goals of cancer informatics are: (1) to organize data in a way that is comprehensible and meaningful to clinicians, researchers, and patients; (2) to use data to advance cancer care and treatment; and (3) to yield new insights through data analysis [2].

The multidisciplinary field of cancer informatics includes oncology, pathology, radiology, computational biology, physical chemistry, computer science, information systems, information management, biostatistics, clinical informatics, bioinformatics, imaging informatics, machine learning (ML), artificial intelligence (AI), data mining, data compliance, and many other disciplines. The integration and intersection of these individual disciplines bridge the gap between these individual cancer‐related fields and promote cancer research and clinical practice.

From the point of view of informatics, methods and tools enhance the classification, accessibility, and applications of oncology data, thereby transforming cancer treatment into better outcomes. For example, with the development of clinical and imaging oncology databases, radiomics and artificial intelligence have flourished, providing clinicians with a technological foundation for the early detection and treatment of cancer. In clinical practice, radiologists are under tremendous pressure as the number of cancer patients increases quickly. Studies in AI radiotherapy aim to make radiotherapy easier and faster and turn this labor‐intensive procedure into a technology‐intensive task. Another example is the multi‐omics analysis of precision oncology. Multi‐omics analyses can effectively overcome the limitations of single omics by integrating the analysis of a large amount of biological data at the molecular level in different dimensions, such as the genome, epigenome, transcriptome, proteome, metabolome, and microbiome. Moreover, it provides multi‐level analyses and interpretations of complex life phenomena with many influencing factors, such as processes and diseases. With the popularization of next‐generation high‐throughput technologies and the accumulation of large amounts of multi‐omics data, integration and fusion analysis for precise diagnosis and treatment of cancer has become an emerging trend.

**Figure 1:**
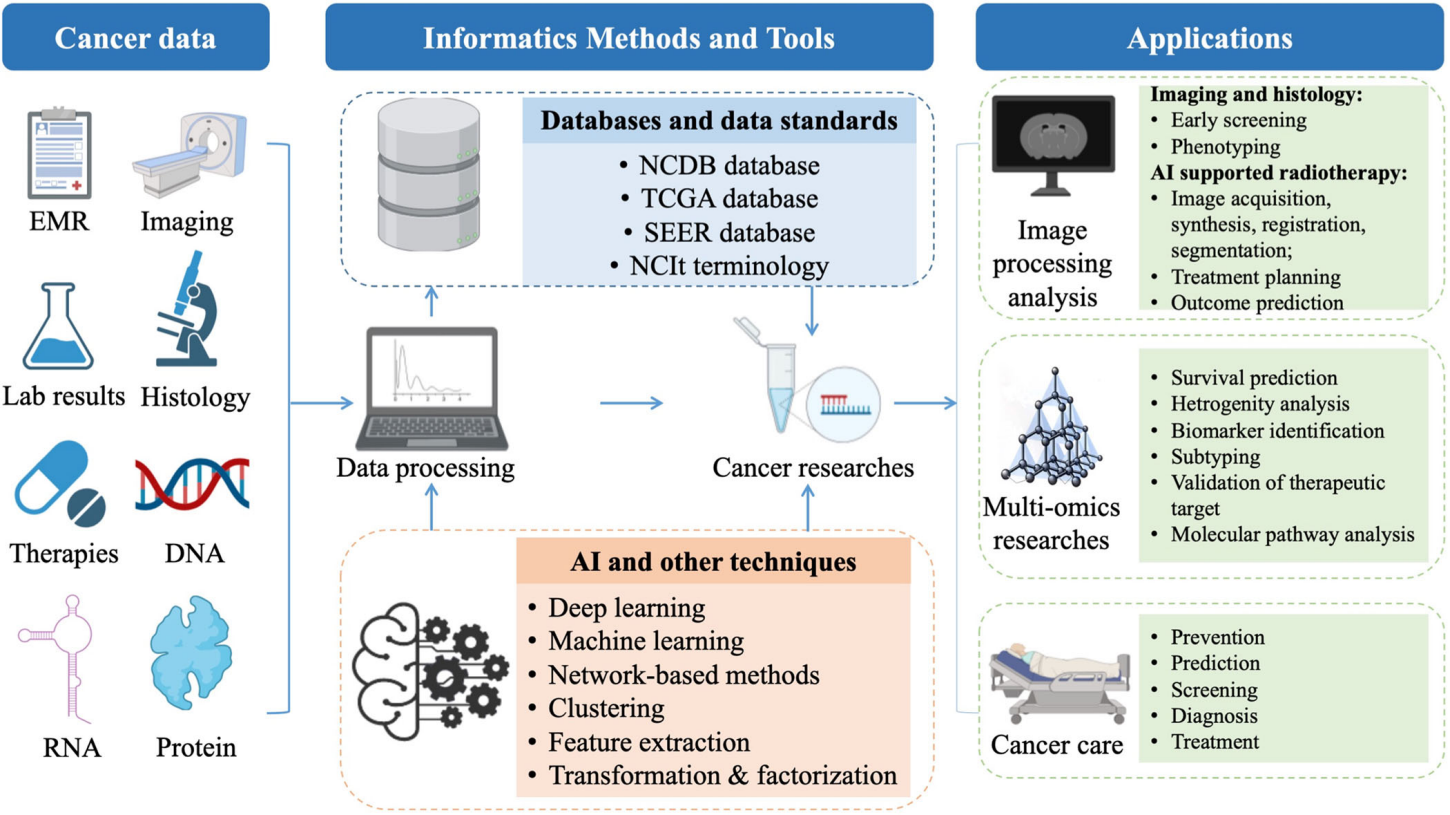
A summary of the main points of cancer informatics. AI, artificial intelligence.

To summarize the current progress in informatics methods and tools to enhance cancer research and improve cancer clinical practices, we reviewed the most common recent scenarios of informatics‐supported applications. A graphic abstract summarizing the field of cancer informatics is depicted in Figure 1.

## 2 INFORMATICS‐SUPPORTED APPLICATIONS OF CANCER RESEARCH AND CLINICAL PRACTICES

Informatics‐based publications are available from the National Library of Medicine database (PubMed) and officially released web resources, which include cancer databases, cancer knowledge organization systems, cancer omics, and precision medicine, as well as AI‐supported cancer imaging and radiotherapy. In this review, retrieved articles were manually screened according to a criterion containing the following items: aim of the study, methods, results, and clinical scenarios.

### 2.1 Databases and data standards for oncology

Healthcare data stored in various electronic systems follow different formats, whether structured or unstructured data. The information contained in medical records contains critical elements that support cancer therapies. Storing, extracting, and encoding such information plays an important role in cancer treatment and research. Population‐based cancer registry databases can record information on incidence, mortality, and treatment outcomes, generating annual statistics as a result [3]. In contrast, hospital‐based cancer databases provide more clinical information than population‐based cancer registries, such as patient information, clinicopathological information, genomic data, disease staging, treatment, follow‐up, lab test results, and medical records, which supports clinical research and improves the care of cancer patients [3, 4]. Furthermore, a consistent system of coding needs to be ensured to integrate the collected data from different sources that could be encoded in various terminological standards [5]. In addition, ontology, as an integration of knowledge, annotation, and concepts, plays an important role in cancer treatment and research.

#### 2.1.1 Cancer databases and scientific programs

The database built by the National Cancer Institute's (NCI's) Surveillance, Epidemiology, and End Results (SEER) program in 1973 and by the Centers for Disease Control and Prevention's National Program of Cancer Registries of the United States in 1995 is used to construct the US Cancer Statistics [6, 7], while data from the National Central Cancer Registry of China is used to produce cancer statistics in China [8, 9]. The National Cancer Database of the United States is one of the largest cancer clinical registry databases, with over 34 million data sets of commonly diagnosed solid tumors added since 1989, and has an increasing number of published studies [10, 11]. Moreover, thousands of new genomes have been sequenced over the past few years [12]. The Cancer Genome Atlas was initiated in 2006 and has characterized more than 20,000 primary cancers at the molecular level, covering 33 cancer types to date. This database consists of genomic, expression, methylation, copy number variation, epigenomic, transcriptomic, and proteomic data with more than 2.5 petabytes in volume [13, 14]. The International Cancer Genome Consortium supports genomic studies in more than 50 cancer types involving more than 25,000 cancer genomes at the genomic, epigenomic, and transcriptomic levels [15].

#### 2.1.2 Cancer classification, terminology, and ontology

Cancer classification is the prime issue during patient treatment. The International Classification of Diseases for Oncology (ICD‐O) published by the World Health Organization is widely implemented for tumor disease classification. ICD‐O uses a multi‐axial coding system to classify the anatomical site and the histology of a tumor. The first, second, and third editions of ICD‐O were published in 1976, 1991, and 2000, respectively [16, 17, 18]. Furthermore, the Systematized Nomenclature of Medicine Clinical Terms (SNOMED CT) uses concepts, descriptions, and relationships to build terminology systems that can map and link to other standards [19, 20]. It is used to encode cancer pathological checklists that aim to provide interoperable and portable diagnostic, prognostic, and predictive elements [21, 22]. The NCI has published a comprehensive logic‐based terminology, the National Cancer Institute Thesaurus (NCIt), covering cancer‐related components, such as clinical findings, drugs, treatments, anatomy, genes, proteins, and molecular information [23]. Adverse events (AEs), a critical element in cancer clinical trials and research, are recorded in dictionaries such as the Common Terminology Criteria for Adverse Events and the Medical Dictionary for Regulatory Activities developed by the NCI and the International Conference on Harmonization, respectively [24, 25]. HemOnc, which contains information on drugs and regimens regarding their mechanism, U.S. Food and Drug Administration (FDA) approval, common usage, and synonyms, is published and maintained to meet the growing number of chemotherapeutic regimens by combining various definitions, such as RxNorm, SNOMED CT, and the National Cancer Institute Thesaurus (NCIT) [19, 26, 27].

Cancer Care Treatment Outcome Ontology (CCTOO) describes treatment or trial endpoints for patients with solid tumors in 4 domains, 13 subgroups, and 2 concept hierarchical structures with a total of 1133 terms [28]. Alternatively, TNM‐Ontology (TNM‐O) consists of four parts: a representation of the primary tumor (T), a representation of regional lymph nodes (N), a representation of distant metastases (M), and the anatomical location of the tumor. It sets different T, N, and M code descriptors for tumors at different anatomical locations. TNM‐O was implemented in a colorectal cancer database and achieved a 100% concordance rate after validation by experienced pathologists [29]. Radiation oncology ontology (ROO) was published using Semantic Web technologies forming a hierarchical structure containing 1183 classes and 211 properties between classes [30], while Radiation Oncology Structures (ROS) ontology was developed using a taxonomic hierarchy consisting of 417 classes, each with a number of subclasses, 81% of which can be mapped to the Unified Medical Language System (UMLS) [31]. Cancer Cell Ontology (CCL) was published to represent cancer cell types via immune phenotypes in the field of hematological malignancies with a total number of 6900 classes (over 300 new classes added) [32]. Prostate Cancer Ontology (PCO) represents integrated information from multiple prostate databases using a nine‐level hierarchical structure with 412 concepts [33] and local terminologies, such as Cervical Cancer Common Terminology [34], which are used for supporting semantic interoperability and utilization of local clinical data.

### 2.2 AI‐supported image processing and radiotherapy

Medical imaging is a useful and important modality for cancer detection, progression monitoring, and prognosis prediction. Radiomics and radiotherapy are the two most focused medical research and application areas advanced by AI. Radiomics refers to converting images into structured, mineable data [35]. Most AI‐supported image applications focus on early screening and diagnosis using machine‐learning methods based on predefined features extracted from medical images [36]. Radiation therapy is a pivotal cancer treatment that has significantly progressed over the last decade due to numerous technological breakthroughs. Traditional radiation therapy workflows identify areas that would benefit from AI, including imaging, treatment planning, quality assurance, and outcome prediction. Many recent studies have shown that the adoption of radiomics and machine learning has paved the way for improved management of radiation therapy patients.

#### 2.2.1 AI imaging and diagnostics

AI has contributed to medical imaging by improving the quality of images and computer‐aided image interpretation and radiomics in most oncology‐related diagnoses, and the application of AI is crucial in radiology for various modalities with improved quality, such as X‐rays, ultrasounds, computed tomography, magnetic resonance imaging (MRI), positron‐emission tomography (PET), and digital pathology. To analyze these quantitative data, data images, predictive models, diagnosis, prognosis, and longitudinal monitoring based on a parsimonious set of informative imaging features are yielded. Images are analyzed with highly specialized algorithms with increased speed and accuracy. According to a number of papers published in recent years, the most common cancer locations are the breast, kidney, brain, lung, prostate, cervix, and liver. The main AI algorithms are Convolutional Neural Network (CNN), Neural networks (NN), Support vector machines (SVM), Deep Neural Networks (DNN), and Ensemble learning techniques [37]. A recent study outlined the development and validation of an automated detection system for chest radiography with algorithms based on deep learning [38]. This automated system is designed to diagnose common thoracic diseases including lung malignancies. The results of this study showed that AI‐integrated systems have superior image recognition and analysis capabilities compared with human observers. For example, mammography is the first line of imaging screening for breast cancer. For younger women with dense breast tissue, ultrasound is the preferred option, and a previous study demonstrated the influence of AI in breast imaging [39]. The authors compared the interpretation of mammography with and without the assistance of AI. Unsurprisingly, radiologists with AI assistance were able to analyze mammography images quicker and more accurately, which is vital for the rapid detection of cancers, and further research directions for AI in medical imaging will focus on improving speed and reducing costs [40, 41]. Previous studies have also reported AI tools developed by Google that can search for morphologically similar features [41], regardless of annotation status. For example, LYmph Node Assistant (LYNA) is Google‐developed deep learning algorithm that can successfully detect metastatic breast cancer on slides with up to 99% accuracy.

#### 2.2.2 AI‐supported radiotherapy

In radiotherapy, images from different patients, times, or modalities often need to be registered to synthesize their corresponding information in a joint coordinate. The registration of images is relatively simple. However, how to achieve the registration of images and pathology (biomarkers) obtained or analyzed by different modalities is a current problem. At present, the prediction of biomarkers according to images does not achieve accurate point‐to‐point matching. A study was conducted to set up the conditional Generative Adversarial Network (cGAN), which uses synthetic computed tomography (sCT) images from low field MR images in the pelvis and abdomen, and compares the differences in dose‐volume histograms between sCT and original CT [42]. Deep learning has been used to improve the quality and efficiency of deformable image registration (DIR) [43]. Given the unavoidable nonrigid anatomical motion by the patient between image acquisitions, DIR needs to establish a voxel‐to‐voxel correspondence between two medical images that reflects these two different anatomical instances [44, 45]. In addition, treatment planning benefits from AI and information technologies. An array of research with dose prediction or validation has been published in recent years. Multiple dose levels, radiation‐sensitive critical structures near target organs, and tumors in the abdomen, head, and neck were the most researched areas among recent achievements [46]. To enable accurate MRI‐based dose calculations, Matteo et al. generated sCT from T1‐weighted MRI using three 2D conditional cGANs [47]. Furthermore, new devices, such as electronic portal imaging devices [48] and kV cone‐beam computed tomography images [49], have reconstructed the 3D dose distribution in radiotherapy treatment. AI also supports radiotherapy outcome prediction, a dual‐input channel hybrid deep learning model that efficiently integrates an entire set of dosimetric parameters for radiation treatment planning, which was developed to enhance the prediction of Grade 4 radiotherapy‐induced lymphopenia [50].

### 2.3 Cancer multi‐omics research

Unlike evidence‐based medicine, studies on precision oncology should be data‐driven, and omics data are among the most critical. Omics is a type of biotechnology that analyzes the structure and function of the overall composition of a given biological function at different levels. With the development of high‐throughput technologies, such as next‐generation sequencing (NGS) and mass spectrometry‐based techniques such as LC‐MS/MS, it is possible to facilitate the investigation of the genome, transcriptome, proteome, and metabolome. Compared with single‐level omics, multi‐omic approaches can reveal the molecular mechanisms underlying different phenotypic manifestations of cancer from multiple dimensions. Thus, multi‐omics has been proposed as the key to precision oncology in clinical practice. Together, these omics data can help to reveal the complex molecular mechanisms in different diseases [51]. Multi‐omics can generate more information, and how to achieve multi‐omics registration deserves further research.

#### 2.3.1 Genomics, proteomics, metabolomics, and microbiomics in cancer research

Scientists have identified several mutated cancer genes through DNA sequencing techniques, such as *PIK3CA*, *EGFR*, and *HER2* [52, 53, 54]. In recent years, the application of NGS for DNA sequencing, coupled with analytical methods, has enabled unprecedented speed and precision in decoding human genomes [55]. In addition, NGS techniques have dramatically reduced the cost of sequencing. Massively parallel sequencing allows further insights into cancer disease from various aspects, including diagnosis, classification, therapeutics, and risk prediction [56]. In addition to differences in gene expression, a study has suggested that DNA methylation, a reversible DNA modification, can be used as an indicator of cancer status [57]. The identification of DNA modifications, including methylation, acetylation, histone modification, and nucleosome remodeling, is defined as epigenomics. These modifications are critical in regulating the biological processes fundamental to cancer genesis [58]. Several factors such as genetic and environmental factors can affect DNA modifications, which might be long‐lasting or even heritable [59, 60, 61]. Hence, epigenomics data has great potential in the interpretation of genetic variants in cancer. Compared with DNA, RNA molecules change temporally according to cellular, environmental, extracellular, and developmental stimulation. The application of NGS has also facilitated transcriptomics studies because we can identify both the presence and abundance of RNA transcripts in a genome‐wide manner via RNA‐sequencing [62]. Studies on transcriptomics have revealed characteristic gene expression signatures in various cancer types that can help in clinical decisions, including diagnosis, treatment choices, and disease management. Furthermore, several clinical trial findings have been applied to predict the prognosis of different cancers, such as breast and lung cancer [63, 64]. Gene expression sequencing has also been extended to single cells, which enriches the data of cancer cells and helps us to understand cancer heterogeneity [65, 66].

In cancer research, proteomics data has contributed to the development of biomarkers in cancer identification as well as classification, prediction of drug sensitivity, and identification of proteins that may mediate drug resistance in different cancer types [67, 68, 69]. The development of LC‐MS/MS techniques has provided a platform for proteomic analysis, for example, supporting proteomic alterations in various cancer tissues. The application of LC‐MS/MS can be extended to small molecules, which allows us to study metabolomics data. Compared with the omics mentioned above, metabolomics is a new field, and most studies of cancer metabolomics have focused on the identification of biomarkers in plasma or serum samples, such as unsaturated free fatty acids in colorectal cancer and citrate changes in prostate cancer [70, 71]. Furthermore, microbiomics data give us brand new insights into cancer research and provide further information on the underlying molecular mechanisms in cancer genesis and development. It is suggested that the dysbiosis of symbiotic microbiota is related to several types of cancer [72]. In addition to cancer triggering or promotion, the microbiome can also be used in cancer therapies, including therapeutic targets and microbiota transplantation [72, 73].

#### 2.3.2 Integrated multi‐omics analysis for precision oncology

The integration and analysis of high‐throughput omics data are complex but critical. Data‐driven methods include deep learning, network‐based methods, clustering, features extraction, transformation, and factorization, which connect the data and clinical and molecular features of cancer [74]. Furthermore, multi‐omics studies on cancer cover many goals, including biomarker discovery, subgroup identification, molecular pathway analysis, and drug repurposing/discovery. Table 1 summarizes some multi‐omics studies conducted on cancer in recent years. These findings have contributed to precision oncology in clinical decision‐making and mechanism studies.

**Table 1:** Examples of multi‐omics studies in cancer

Article	Objective	Cohort/database	Omics data				
			Genomics	Epigenomics	Transcriptomics	Proteomics	Metabolomics
Chaudhary et al. [75]	Survival prediction	TCGA + multiple center	☑	☑	☑		
Zhang Q et al. [76]	Analysis of tumor heterogeneity	Single center	☑	☑	☑	☑	☑
Seal DB et al. [77]	Estimating gene expression	TCGA		☑	☑		
Ouyang X et al. [78]	Biomarker identification and subtyping	TCGA + GEO		☑	☑		
Huang G et al. [79]	Establishing a model for survival prediction	TCGA	☑	☑	☑		
Löffler MW et al. [80]	Validation of therapeutic target	Single center	☑		☑	☑	
Shen M et al. [81]	Analysis of molecular pathways in HCC cell	HepG2 cell line			☑		☑

Abbreviations: GEO, Gene Expression Omnibus; TCGA, The Cancer Genome Atlas.

#### 2.3.3 Biomarker identification for cancer prevention, diagnosis, and prognosis

Molecular biomarkers identified from omics data are often used for cancer prevention and diagnostics by detecting early disease. Cancer surveillance can be improved by identifying clinically relevant biomarkers for the early prevention of disease and to predict prognosis for effective treatment, such as carcinoembryonic antigen to monitor the recurrence of colorectal cancer [82, 83] and mutations in estrogen receptor 1 (ESR1) to predict prognosis and treatment outcomes in breast cancer [84]. Furthermore, shallow sequencing has recently been applied to the whole genome for diagnostics in breast cancer [85], lung cancer [86], and neuroblastoma [87].

## 3 CHALLENGES

Driven by electronic and smart technologies, patient data are being generated at an increasingly rapid rate. However, regardless of the benefits of informatics, there are still many barriers to implementing AI in healthcare.

### 3.1 Data heterogeneity

The heterogeneity of cancer data is the primary difficulty in effectively integrating, searching, and extracting information, while the realization of its interoperability is a prerequisite for the implementation of personalized and precise treatment. Therefore, the inability to exchange information between cancer diagnosis and treatment systems becomes a limitation in pursuing data‐driven clinical practice. Developing a global system to formalize and harmonize each individual data model, classification, thesaurus, vocabulary, terminology, and ontology from different systems is the main challenge.

### 3.2 Lack of good governance and annotated data

The limitations of most existing applications are the lack of quality control, data standardization, and sufficient samples. Most radiomics studies use images obtained from a wide range of scanning devices (e.g., CT, MRI, and PET) produced by different manufacturers. The absence of standardized protocols leads to significant variability in data acquisition and reconstruction parameters. Hence, numerous technical problems must be considered, and approved methodologies are needed to distinguish signal from noise in medical images [36], which requires the standardization of image preprocessing, tissue segmentation, feature calculation, and statistical methodologies.

### 3.3 Varying maturity of different informatics approaches in clinical application

The major challenge of informatics applications in cancer is the varying maturity of the different approaches. Genomics has been used for diagnosis, while other omics approaches such as epigenomics and proteomics are less used in clinical practice [88]. The time it takes to run the samples and the equipment requirements for omics data analysis techniques are variable. The technical maturity ranks from high to low as follows: RNA, epigenomics, transcriptomics, metabolomics, and proteomics. Furthermore, although data‐driven analysis in cancer research is rapidly on the rise, most studies have focused on common cancer types, and there is still a lack of investigation of rare or challenging tumor types [89]. Imaging analysis technologies and tools are more mature than clinical data modeling and omics data analysis.

### 3.4 Model generalizability, results interpretation, and external validation

The generalizability of machine learning models is challenging. Different image acquisition equipment, different contrast agents, and different image acquisition parameters of the same equipment may have a large impact on the results. Furthermore, another challenge lies in the interpretation of data‐driven results. Current AI predictions are more of a black box, and their interpretability and application are questioned by clinicians and require attention. This also brings challenges to the promotion of devices such as intelligent diagnosis. The data generated is only useful when it is clinically relevant and correctly interpreted. Thus, prospective clinical trials are urgently needed. All prospective studies with external validation are needed to translate these results from bench to bedside. However, both the scarcity of external data and the nonuniform method of external validation make this challenging [90, 91]. Furthermore, to implement AI‐based systems for routine clinical practice, the intended users require training and understanding of the system [92].

### 3.5 Cost challenge

The implementation of informatics, AI, and data engineering, such as big data storage, curation, annotation, AI model training, and deployment, requires enormous infrastructure, strong computing power, large storage capacity, massive multidisciplinary specifics, and time to integrate and interpret patient data. Informatics‐supported application systems can be expensive because of their dependence on specialized computational requirements for fast data processing and rich medical knowledge for supporting medical applications appropriately. It is expected that advanced informatics methods and tools will reduce the cost, increase the speed of high throughput data analysis, provide data services in a cost‐ and time‐effective manner, and become widely accessible for cancer research and clinical applications.

### 3.6 Compliance challenge

Informatic systems process a huge amount of patient data, which could trigger the laws and regulations of data security for personal data protection, for example, the Personal Information Protection Law and Data Security Law. How to protect patients' data and process sensitive data efficiently for the purposes of research as well as for clinical application is a challenge for medical institutions. Thus, a systematic approach for the purpose of compliance may apply to informatics practice.

## 4 OPPORTUNITIES AND FUTURE PERSPECTIVES

Cancer burden is a global phenomenon. The reduction of mortality rates requires early diagnosis and effective therapeutic interventions. However, metastatic and recurrent cancers develop drug resistance. Thus, it is imperative to detect novel biomarkers that induce drug resistance and to identify therapeutic targets to improve treatment effects. Informatics methods and tools can be applied to several clinical applications, which are important for risk prediction, early detection of disease, diagnosis by sequencing and medical imaging, accurate prognosis, biomarker detection, and identification of therapeutic targets for novel drug discovery.

As a hierarchical structure with standardized concepts, data standards such as vocabularies, terminologies, and ontologies can promote tumor data integration in many aspects. As an easier and faster way to integrate and encode different data systems, vocabulary sharing and ontology matching can promote data communication between scientists and enable rapid information dissemination, thus facilitating the long‐term evaluation of tumor treatment and research. A shared vocabulary standardizes the definition of data elements, which can make both humans and computers readable and accurately transmit information between systems and humans. Meanwhile, the semantic relationship between the data elements in an encoded system can also support the derivation of conclusions. Furthermore, ontology matching would entail establishing the relationships that exist between the terms of different ontologies. Therefore, it is beneficial to develop automatic mapping algorithms and ensure semantic consistency.

In addition, the application of high‐throughput multi‐omics data and mass spectrometry enable cancer researchers to perform large‐scale studies to analyze the cellular/disease progression of various dimensions, from genome to proteome and metabolome. Furthermore, advanced methods and powerful computational tools will help to identify the links between the phenotypes and omics data. Multi‐omics data platforms provide an opportunity to better understand cellular pathways in disease processes. Genomic analysis in cancer research has made significant progress in recent decades, and further studies will focus on RNA, protein, and metabolite changes and the role of the microbiome in disease. This systematic research on multi‐level data can promote the development of prediction models and practical strategies for personalized cancer therapy [62].

AI techniques, particularly ML, have been extensively applied to process large‐scale and heterogeneous cancer data. These techniques have achieved good results in data mining and analysis by providing powerful algorithms. Therefore, future studies on cancer will be based on AI techniques to process not only structured clinical data but also other unstructured clinical data, such as electronic medical records, imaging, and omics data. AI has made a significant impact and will continue to revolutionize healthcare and precision oncology. Considering the interdisciplinary nature of cancer informatics, the collaboration of multiple disciplines is a major driver for future research and applications.

## 5 CONCLUSION

In conclusion, clinical oncology and research are reaping the benefits of informatics. Using informatics methods and tools, a large amount of diverse and dynamic data plays an important role in cancer research and clinical practices in the workflow of data collection, modeling, interoperability, integration, analysis, and utilization. With the further development of convenient and intelligent tools, informatics will enable earlier cancer detection, more precise cancer treatment, and better outcomes.

## AUTHOR CONTRIBUTIONS

**Na Hong**: conceptualization (equal); formal analysis (equal); investigation (equal); writing – original draft (equal); writing – review and editing (equal). **Gang Sun**: conceptualization (equal); formal analysis (equal); methodology (equal); writing – original draft (equal). **Xiuran Zuo**: investigation (equal); methodology (equal); resources (equal); writing – original draft (equal). **Meng Chen**: methodology (equal); writing – review and editing (equal). **Li Liu**: methodology (equal); writing – review and editing (equal). **Jiani Wang**: investigation (equal); writing – review and editing (equal). **Xiaobin Feng**: methodology (equal); writing – review and editing (equal). **Wenzhao Shi**: funding acquisition (equal); supervision (equal). **Mengchun Gong**: conceptualization (equal); funding acquisition (equal); project administration (equal). **Pengcheng Ma**: conceptualization (equal); funding acquisition (equal); writing – original draft (equal).

## CONFLICTS OF INTEREST

Professor Meng Chen and Mengchun Gong are members of Cancer Innovation Editorial Board. To minimize bias, they were excluded from all editorial decision‐making related to the acceptance of this article for publication. The remaining authors declare no conflict of interest.

6

## ETHICS STATEMENT

The authors are accountable for all aspects of the work in ensuring that questions related to the accuracy or integrity of any part of the work are appropriately investigated and resolved. This paper is the authors' own original work, which has not been previously published elsewhere. And it reflects the authors' own research and analysis in a truthful and complete manner. The paper properly credits the meaningful contributions of coauthors and coresearchers. This article does not contain any studies with animals performed by any of the authors.

## INFORMED CONSENT

Not applicable.

## Data Availability

Data sharing is not applicable to this article as no new data were created or analyzed in this study.
